# Collaborative Robot Control Based on Human Gaze Tracking

**DOI:** 10.3390/s25103103

**Published:** 2025-05-14

**Authors:** Francesco Di Stefano, Alice Giambertone, Laura Salamina, Matteo Melchiorre, Stefano Mauro

**Affiliations:** Department of Mechanical and Aerospace Engineering, Politecnico di Torino, 10129 Torino, Italy; s288086@studenti.polito.it (A.G.); laura.salamina@polito.it (L.S.); matteo.melchiorre@polito.it (M.M.); stefano.mauro@polito.it (S.M.)

**Keywords:** gaze tracking, collaborative robotics, human–machine interaction, robot control, eye tracking technologies

## Abstract

Gaze tracking is gaining relevance in collaborative robotics as a means to enhance human–machine interaction by enabling intuitive and non-verbal communication. This study explores the integration of human gaze into collaborative robotics by demonstrating the possibility of controlling a robotic manipulator with a practical and non-intrusive setup made up of a vision system and gaze-tracking software. After presenting a comparison between the major available systems on the market, OpenFace 2.0 was selected as the primary gaze-tracking software and integrated with a UR5 collaborative robot through a MATLAB-based control framework. Validation was conducted through real-world experiments, analyzing the effects of raw and filtered gaze data on system accuracy and responsiveness. The results indicate that gaze tracking can effectively guide robot motion, though signal processing significantly impacts responsiveness and control precision. This work establishes a foundation for future research on gaze-assisted robotic control, highlighting its potential benefits and challenges in enhancing human–robot collaboration.

## 1. Introduction

Collaborative robotics is a rapidly evolving field that is transforming the interaction between humans and machines in shared workspaces, improving productivity, safety, and efficiency across various applications. This evolution encompasses the development of new control algorithms, as well as the introduction of a new category of robots, known as Collaborative Robots (COBOTs). Unlike traditional robots, which replace humans in physically demanding or high-precision tasks, cobots are designed to work side-by-side with humans, so that they can perform a wide range of functions, especially in processes that cannot be fully automated. To ensure the safety and ergonomics of the task, cobots are designed so that exposed mechanical parts, such as joints, links, and the gripper, are smoothed and integrate force, torque, or contact sensors.

The collaboration can be either physical, when involving intentional contact and force exchange between humans and robots, or contactless, when they carry out a task in the shared workspace without direct physical interaction. An example of physical collaboration is the hand-over, since it is a process of exchanging objects or information between a giver and a receiver. Most of the previous research on human–robot hand-overs has developed an offline approach, requiring human adaption to the robot’s predetermined actions [1,2]. Online strategies have also been developed that aim to improve the dynamic response of the robot to real-time data and the adaptation of human actions during hand-over. An example can be seen in Wang et al.’s study [3], in which a wearable sensory system captured the motion information of a human’s arm and hand, which was subsequently processed to recognize the human’s hand-over intentions. Alternatively, the authors’ previous work [4] used a camera system to track the movement of the operator’s arm for a more responsive hand-over. A typical contactless collaboration, on the other hand, usually involves collision avoidance algorithms. In [5], this was implemented using potential fields, where a repulsive field was assigned to the human detected by the vision system. The system tracked the operator’s movements and adjusted the robot’s trajectory accordingly.

Improving human–machine interaction to more closely resemble human–human collaboration is a key objective in the field of collaborative robotics. Human coordination involves both explicit communication, such as verbal commands, and implicit cues like intention recognition [6,7]. Among non-verbal modalities, such as gestures, body language, and eye contact, gaze plays a particularly crucial role, as it reflects a natural cognitive process that often anticipates physical action [8,9]. Unlike gestures, which require predefined mappings and conscious execution, gaze is typically involuntary and provides insight into the user’s intent [9]. This contributes to more fluid and coordinated interaction, similar to that found in human–human collaboration. For these reasons, gaze tracking was selected as the focus of this study, as it represents a promising approach to improve the synchrony and intuitiveness of collaborative tasks such as hand-over and shared manipulation [7,10,11].

The use of gaze in human–robot interaction has already been studied. The article in Ref. [12] gives an overview of possible applications and points out the importance of referential gaze—i.e., gaze understood as an indication of a point or area of interest in space—in collaborative robotics. For example, in [13], the direction of gaze, tracked via a wearable system consisting of a camera and a Magnetic AngularRate Gravity (MARG) sensor, was used to control the movement of a system consisting of two UR5 arms. In [14], gaze direction, tracked via wearable glasses, was used to identify points in space to which a Kinova MICO should be directed, and the importance of gaze in anticipating a person’s intentions and speeding up task performance was emphasized. Furthermore, [15] proposed the use of augmented reality glasses to guide a robot, in this case a mobile one. However, these works rely on wearable systems that compromise the ergonomics of the task. A work that relied on the use of an external camera was [16], in which objects were located that a person pointed at using their gaze. Our work differs from the aforementioned research, since it aims to use referential gaze to control the robot in real time using an external camera. To achieve this, we do not simply identify a possible area of interest in the workspace, but use point information derived from the gaze vector calculated by gaze-tracking software.

The innovation introduced by this study lies in the formalization of a simple and easily replicable control framework, both in terms of hardware and software. The system is built using OpenFace 2.0, an open-source gaze-tracking tool, and MATLAB, a widely adopted platform in research environments for algorithm implementation and testing. The overall architecture can be adapted to different acquisition tools, meaning that components such as the gaze-processing module can be replaced, if needed, without altering the main control logic. This flexibility enables researchers and developers to adapt the system to different use cases or available technologies. This work may serve as a practical guide for those approaching the integration of gaze tracking in robotic control systems and is inspired by the authors’ previous work [4]. The article provides a comprehensive overview of various gaze-tracking technologies, highlighting the existing categories, their differences, and their strengths. Therefore, an algorithm is presented to control the robot so that it moves in the direction of the operator’s gaze. In addition, efficient software implementation is described, which enables dynamic gaze tracking by the robot. The algorithm uses a MATLAB-based interface that bridges the robot’s control system with the gaze-tracking software through a dual-loop structure. The first loop ensures the creation of a file containing gaze data, while the second loop retrieves and processes this data, verifying its uniqueness and proper integration. The validation of the approach was conducted through real-world experiments with a UR5 collaborative robot. The experiments utilized both raw gaze data and data processed using a moving average filter to improve stability and optimize performance, in order to enhance the balance between responsiveness and accuracy in human–robot interactions.

In the following section, a comprehensive overview of the main aspects of gaze-tracking systems and their subdivisions is presented.

## 2. Gaze-Tracking System

Gaze tracking monitors and records eye movements, determining where a person is looking, for how long, and in what sequence, to infer their needs and interests in the surrounding environment [17]. Gaze-tracking methods can be intrusive or non-intrusive. Intrusive systems use wearable devices, such as specialized glasses with embedded sensors, offering high accuracy but potentially interfering with user’s natural behavior, as these devices can be uncomfortable to wear and may restrict or alter the user’s normal movements and actions. Non-intrusive systems, on the other hand, rely on external cameras to track eye movements. As technology advances towards intelligent systems, the emphasis has shifted from solely accuracy to also enhancing user experience. Consequently, non-intrusive gaze-tracking systems, known as Remote Eye Gaze Trackers (REGTs), have become the preferred choice [18]. REGTs prioritize user comfort and mobility, making them particularly suitable for scenarios where wearing specialized devices would be inconvenient or impractical, such as in collaborative robotics [17,19].

In REGT, two main computational approaches can be distinguished: feature-based and appearance-based methods. Feature-based methods estimate gaze direction by extracting local features, e.g., pupil and glints, from the eye region using computer vision techniques. These methods remain the predominant approach for gaze estimation [18]. Appearance-based methods, on the other hand, analyze the overall visual representation of the eye region rather than focusing on specific features. These approaches use images captured by visible light RGB cameras, and then employ machine or deep learning algorithms that directly regress on these images for gaze estimation. During training, subjects fixate on predefined screen locations, allowing the model to learn the relationship between eye appearance and gaze direction. This enables the system to predict gaze based on observed patterns [18,20]. Common algorithms for feature extraction and gaze point mapping include genetic algorithms, Bayesian classifiers, support vector machines, and artificial neural networks [18].

Despite extensive research, gaze tracking remains challenging due to factors such as eyelid occlusion, eye size variability, reflectivity, and head pose. These complexities make it difficult to find a single, cost-affordable solution that addresses all issues comprehensively. Various devices and software solutions are available for gaze tracking, each with specific advantages and limitations. Among the open-source tools evaluated, GazeTracking [21] is a Python library that provides a webcam-based eye-tracking system, capable of detecting pupil position and gaze direction in real time. OpenFace 2.0 [22,23] is a versatile tool that provides facial landmark detection, head pose estimation, facial action unit recognition, and eye-gaze estimation. It supports real-time output, data saving, and streaming over a network, while being compatible with simple webcams. GazeSense [24], developed by Eyeware, enables the real-time tracking of eye movements and gaze direction, supporting integration with both 3D cameras and webcams. Pupil Core [25,26] is an open-source eye-tracking platform that provides real-time pupil detection, gaze mapping, and data visualization. It requires a dedicated Pupil Labs headset with high-resolution cameras, or other UVC-compatible cameras, for tracking. Lastly, GlassGaze [27] is a client for the open-source Haytham gaze tracker, specifically designed to facilitate eye and gaze tracking on Google Glass.

Commercial eye-tracking systems were evaluated too, as they combine both hardware and software components to provide cohesive and efficient eye-tracking results. The Tobii Pro Glasses 3 [28] is a wearable eye tracker designed to capture the view while providing robust and precise eye-tracking data. It supports live view of the scene via camera video and provides additional data channels, such as two-dimensional (2D) and three-dimensional (3D) gaze, gaze origin, gaze direction, and pupil diameter. On the other hand, the Tobii Pro Spark [29] is a compact, high-performance screen-based camera that uses sophisticated image processing algorithms to identify key features such as eyes and corneal reflection patterns. With its dedicated software, Tobii Pro Lab and Tobii Pro SDK, it enables the recording, analysis, and visualization of eye-tracking data, including 3D eye coordinates, raw data, and pupil measurements. The SMI Eye Tracking HMD [30] is another noteworthy system, designed for the Oculus Rift DK2. This system provides real-time eye and gaze data and is accompanied by a C/C++ SDK for integration. Lastly, the Smart Eye Pro [31] is a multi-camera system, scalable from two to eight cameras, enabling 360-degree head and eye tracking. It also includes an SDK for integrating eye tracking capabilities into other products and applications.

In Table 1, the principal characteristics of the presented gaze-tracking systems are shown. In a preliminary study, the integrated systems were evaluated as unsuitable due to their high cost. Conversely, OpenFace 2.0, although exhibiting a higher error rate, provides an open-source platform primarily in MATLAB. It also provides a high operating distance, making it the preferred choice.

**Figure 1:**
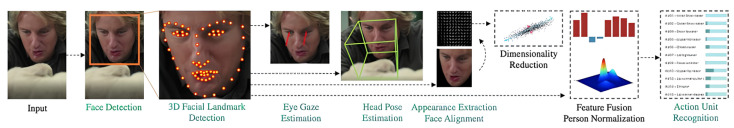
The OpenFace 2.0 pipeline encompasses landmark detection, head pose and gaze estimation, and facial action unit recognition. The outputs highlighted in green can be stored on disk or transmitted over a network in real time. Adapted from [22].

Figure 1 illustrates the OpenFace 2.0 processing pipeline. The software identifies facial keypoints using two main components: the Constrained Local Model, which captures general facial variations, and Convolutional Experts, a convolutional neural network that enhances recognition by focusing on specific facial regions.

For eye-gaze estimation, a Constrained Local Neural Field landmark detector identifies eye landmarks. The algorithm projects a ray from the camera origin through the pupil center in the image plane, computing its intersection with the modeled eyeball sphere to determine the pupil’s 3D coordinates. The gaze vector is then estimated as the direction from the eyeball center to the pupil location. The gaze vector data consist of the gaze direction components along the principal axes of the camera frame for both eyes. Additionally, the software provides an averaged gaze angles along the x and y axes, corresponding to left–right and up–down gaze movements, respectively. OpenFace 2.0 can be integrated into C, C++, and MATLAB projects and is compatible with any camera. The output data are stored in a CSV file, with each row corresponding to a processed frame.

## 3. Control Methodology

The focus of this study is to incorporate gaze tracking into the control system of a cobot and evaluate its effectiveness through a simple but representative task. The selected task involves directing the end-effector of a UR5 cobot, combined with a OnRobot RG2 tool [32], to a target position. A participant is positioned in front of a camera, and the captured images are processed using OpenFace 2.0 to extract gaze position data. The output is to obtain the robot following the position of the operator’s point of focus.

The robot control algorithm is based on the target-following algorithm presented in [33], which enables the driving of the robot end-effector towards a dynamic target. In this case, the target is a virtual point in the robot workspace, which is computed accordingly to the direction of the human gaze. In particular, the algorithm receives gaze angles data from the gaze-tracking system and robot joint positions as a feedback from the robot controller. Thus, the target-following algorithm is used to compute the vector of joint velocities for the manipulator, based on the current distance between the end-effector and the virtual point. The vector of joint velocities is then sent as control input to the robot controller. It is important to highlight that the developed system is responsible only for sending target commands to the UR5 robot. The execution and validation of these commands are then handled by the robot’s internal control unit, which ensures the proper management of the received inputs. As a result, all of the robot’s built-in safety features remain active, stopping motion in cases of unexpected contact forces and preventing overloads. The subsequent sections provide a detailed analysis of the primary components of this control strategy.

### 3.1. Retrieval of Gaze-Tracking Data

The first step was the calibration of the implemented camera, the Orbbec Astra Pro, using the ChArUco technique [34] to determine its intrinsic parameters. The gaze-tracking code developed in MATLAB served as an interface between user requirements and the functionalities of OpenFace 2.0. The software enables the configuration of parameters including the camera device number, intrinsic calibration, and output directories. To ensure dynamic functionality, immediate data accessibility, and accuracy verification, a dual-loop mechanism was implemented. The decision to employ two distinct loops was a deliberate optimization strategy aimed at reducing redundant verification checks once the file was detected.

The initial loop was designed to verify the creation of the OpenFace 2.0 .CSV output file, continuously monitoring the specified file path to confirm its existence. The system remained in standby until the file was successfully generated in the predefined output directory.

Once the file creation was confirmed, the system proceeded to the second loop, where the focus shifted to the dynamic retrieval and real-time processing of data. This phase was implemented to prevent any timing inconsistencies between MATLAB’s execution speed and the writing speed of the .CSV document. The system ensured the accurate reading of the last processed line in the .CSV file, verifying its uniqueness and avoiding the accidental inclusion of duplicate data. During this loop, the system extracted and stored the gaze angles, discarding unimplemented data present in the .CSV file.

### 3.2. Gaze-Following Algorithm

**Figure 2:**
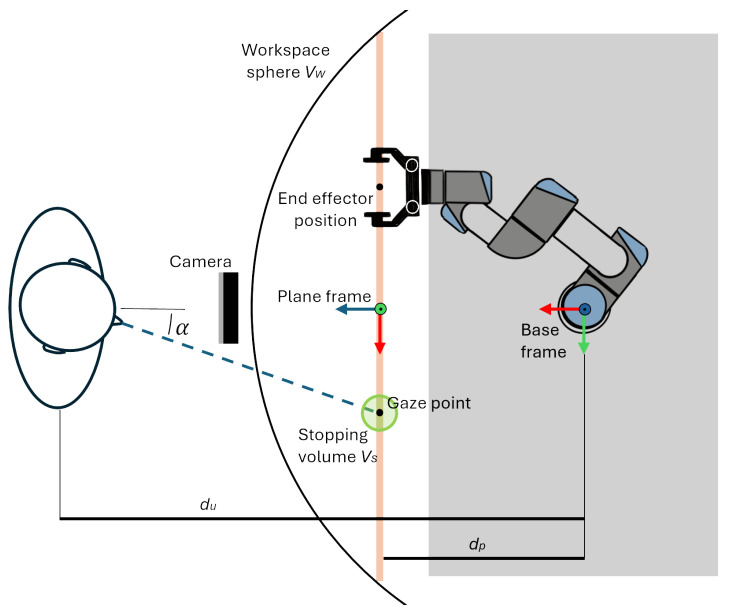
Top-view schematic of the setup. Both the base and plane reference frames shows the *x*-axis in red, the *y*-axis in green, and the *z*-axis in blue. The gaze_angle_x (α), the user’s distance from the base frame ($d_{u}$), and the plane’s distance from the base frame ($d_{p}$) are shown. Additionally, the workspace sphere $V_{s}$ and the stopping volume $V_{p}$ around the gaze point are visible.

This section of the controller converts gaze-tracking data into robot-positioning commands. The gaze vector is obtained by combining the gaze angles provided by the software with the user’s position, which serves as the application point of the vector and is located at a distance $d_{u}$ from the robot’s base frame. Although OpenFace 2.0 provides gaze directions for both eyes, gaze angles were used instead, as they proved more stable in preliminary tests. As shown in Figure 2, gaze_angle_x (denoted as α) represents the angle between the user’s gaze direction and the plane’s *z*-axis, projected onto the *x*-axis. Similarly, gaze_angle_y is the corresponding angle projected onto the *y*-axis. Given the application point and direction, the gaze vector can be determined. However, to define a unique gaze point, a virtual plane was introduced to constrain the end-effector’s movement. The intersection of the gaze vector with this plane defines the gaze point, which serves as the reference for the end-effector’s position.

The position of the plane was defined referring to the base frame of the robot. First, an offset $d_{p}$ along the *x*-axis of the base frame was imposed between the plane origin and base frame origin. This distance was tuned to achieve a balance between the reachability of the end-effector and the ease of movement execution while avoiding singularity conditions. Finally, the reference frame of the plane was oriented so that the *y*-axis points upward, and the *z*-axis was normal to the plane and directed towards the camera.

By knowing the distance of the user from the plane, it is possible to compute the position of the gaze point using the trigonometric relations in Equation (1),as below, with respect to the plane frame.

(1) $$\left\{\begin{array}{c} \left(d_{u}-d_{p}\right)\cdot tan\left(gaze\_angle\_x\right)=x_{gaze}\\ \left(d_{u}-d_{p}\right)\cdot tan\left(gaze\_angle\_y\right)=y_{gaze} \end{array}\right.$$

The inputs of this control phase are the gaze angles and the end-effector position. The control computes the gaze point position and the required velocities at the end-effector. The linear velocities $v_{ee}$ of the end-effector are imposed as function of the position error e between the end-effector position $p_{ee}$ and the gaze point $p_{g}$, as shown in Equation (2) below:

(2) $$v_{ee}=f\left(e\right)=f\left(p_{ee}-p_{g}\right)$$

**Figure 3:**
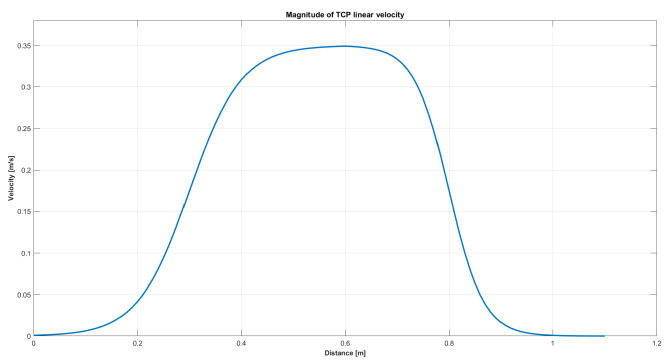
Profile of the end-effector movement when reaching the position target.

Figure 3 shows that the end-effector’s velocity profile was shaped like a bell curve to ensure smooth motion. Additionally, the maximum velocity was constrained to 0.35 m/s.

**Figure 4:**
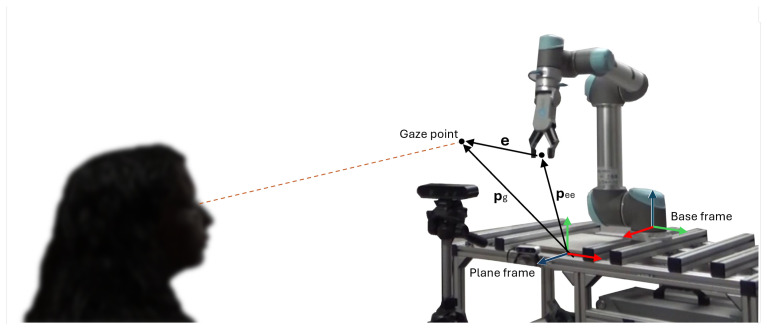
A 3D schematic of the set-up.

In Figure 4, the set-up is shown in a 3D perspective.

The angular velocities $\omega_{ee}$ of the end-effector are set to keep its orientation unchanged. The relation is defined in Equation (3) below:

(3) $$\omega_{ee}=K\cdot e_{o}$$

where *K* is a positive definitive matrix and $e_{o}$ is the quaternion difference between the desired end effector orientation and the actual one. The output of this phase consists of the joint velocities $\dot{q}$ required to reach the target, which are computed using Equation (4), as follows:

(4) $$\dot{q}=J^{-1}\left[v_{ee};\;\omega_{ee}\right]$$

where *J* is the Jacobian matrix. To determine the Jacobian matrix, the robot’s geometry must be known, along with its Denavit–Hartenberg (D–H) parameters. The robot used in this study is the UR5 by Universal Robots, a six-joint anthropomorphic manipulator featuring a non-spherical wrist configuration. The D–H parameters were provided by the manufacturer and can be found in the official documentation [35].

Finally, for the implementation of the control algorithm, the following two spheres were defined:

- The workspace sphere, $V_{w}$, centered on the shoulder of the robot. When the gaze point enters into the range of the sphere, the robot starts moving towards the target.
- The stopping volume $V_{s}$, a virtual sphere around the gaze point that allows the robot to stop when the end-effector enters this volume.

Data processing and joint velocities computation were carried out in MATLAB. Communication between the computer running MATLAB and the UR5 controller was handled via TCP/IP protocol, enabling the real-time transmission of motion commands to the robot. Information sent from UR5 to the computer were encoded in batches of 1220 bytes, which were encrypted as reported in [35]. Information sent from the computer to the UR5 consisted of encrypted strings that corresponded to predefined UR5 commands, along with the required parameters [36].

Algorithm 1 recaps the operating principle of the control script implemented in MATLAB.
**Algorithm 1:** Pseudocode of the gaze-tracking control strategy.
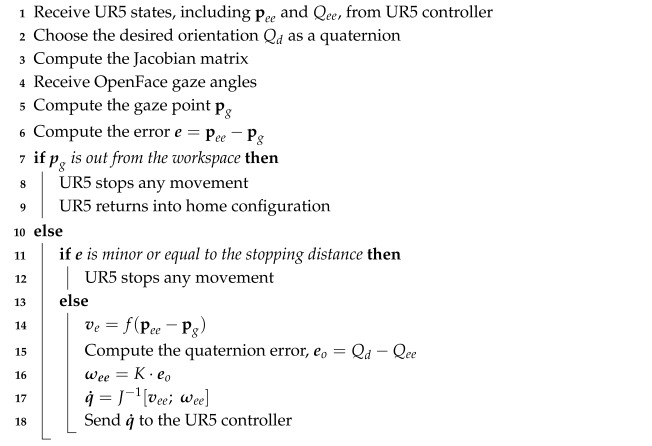


### 3.3. UR5 Controller

The robot control unit manages the operation of the manipulator and facilitates communication with external devices. It houses the necessary electronics for motion control, as well as the software required to process input signals and translate them into actions. The connection between the control unit and external components is established through communication sockets. To ensure reliable communication, the robot must be assigned a static IP address. The robot provides feedback on the instantaneous joint positions, acquired through integrated sensors. Using forward kinematics, the real-time position of the end-effector can be determined.

### 3.4. Full Controller Structure

**Figure 5:**
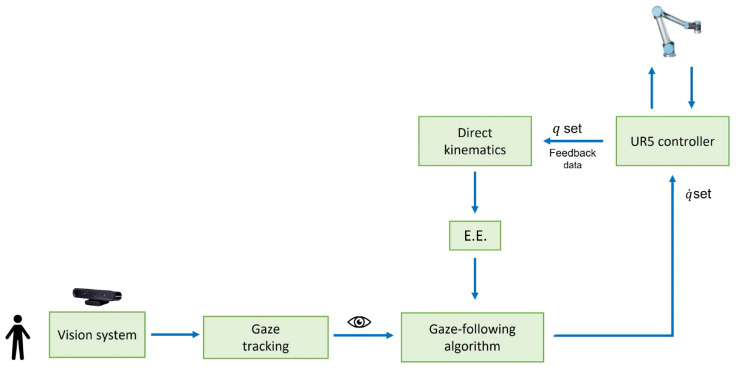
Scheme of the control loop adopted to manage the UR5 robot’s movements.

The final structure of the controller is presented in Figure 5.

The camera works with a frame rate of 30 Hz, while OpenFace expands the images and returns the gaze information at 10 Hz [23]. Gaze data are transferred by OpenFace into a .CSV file, while the MATLAB script takes gaze data from the .CSV file and updates velocity commands to the robot at 2 Hz. This is mainly due to the low speed at which MATLAB reads the data from the .CSV file, as the algorithm for calculating the velocity commands takes only 20 ms. The UR5 controller runs at 125 Hz, and feedback data are retrieved with the same frequency through TCP/IP network communication.

## 4. Results and Discussion

The experimental setup consisted of a computer, a UR5 robotic arm, an Astra Orbbec camera, and a router. The computer functioned as the high-level robot controller, handling data acquisition from the camera, processing feedback from the UR5 controller, and transmitting movement commands to the robot control unit. Table 2 reports the main set-up information.

**Figure 6:**
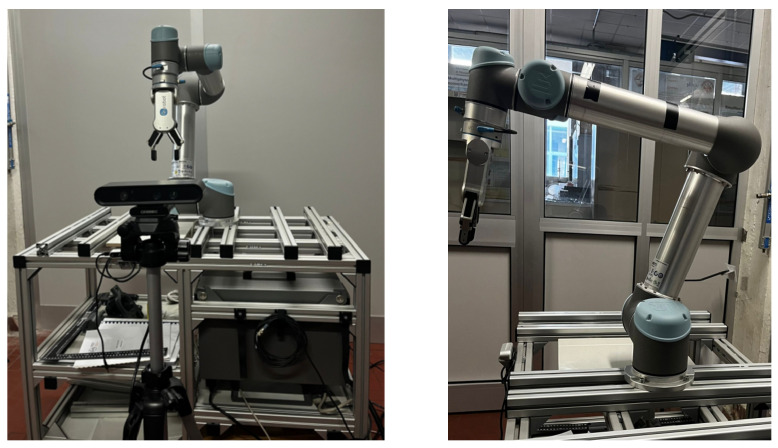
Experimental setup: the UR5 manipulator, in its home configuration, fixed into the workbench, with the Astra Orbbec camera in front of it.

The UR5 base was mounted on a workbench, while the camera was positioned 1.3 m in front of the manipulator, aligned with the robot’s base. The experiments were conducted with the operator seated 0.8 m from the camera to ensure a standardized testing environment. At the start of each trial, the UR5 was set to its predefined home configuration, with the joint angles initialized at [−180°, −70°, −100°, −90°, 90°, and 0°]. The setup can be seen in Figure 6.

The robot remained in its home position until the gaze virtual point entered its spherical workspace, with a radius of 750 mm [37]. Upon detecting the gaze point within this range, the manipulator initiated motion, tracking the target by moving the end-effector to the indicated position. The movement ceased when the distance between the end-effector and the gaze point reached the stopping volume, i.e., when the distance reached 40 mm. If the gaze point moved beyond the workspace boundary, the UR5 returned to its home configuration.

Three experimental conditions were tested. In the first case, the raw gaze signal was used without being processed. In the second and third tests, the signal was filtered using a moving mean filter, in order to reduce noise and smooth random fluctuations in time-series data. This filtering method operates by continuously updating the average within a shifting window along the time series. Throughout all trials, the operator moves their gaze in an attempt to make the robot move along a rectangular trajectory, so as to sweep the virtual plane in height and length.

### 4.1. Original Gaze Signal

**Figure 7:**
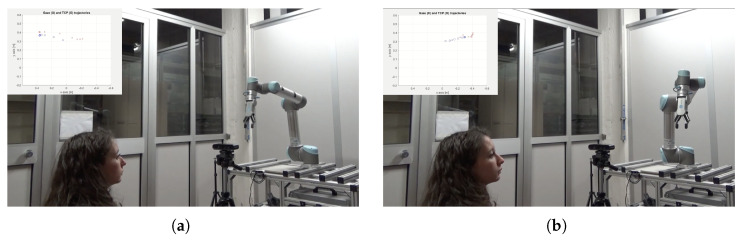
Frames extracted from the video found in the Supplementary Materials. Red crosses indicate the gaze point position and blue circles indicate the end-effector position. (**a**) The user initiates a change in gaze direction. (**b**) The user fixates on a specific point, waiting for the robot to reach it.

**Figure 8:**
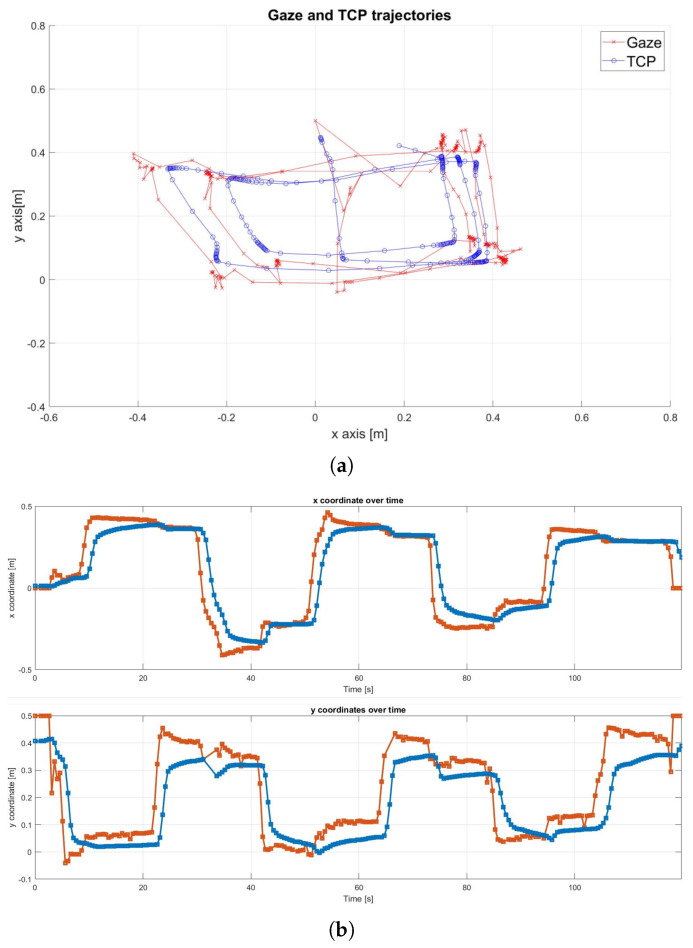
Original gaze signal, wherein gaze is represented in red and the end-effector is represented in blue. (**a**) Trajectories in the x-y plane. (**b**) Trajectories along the x and y axes over time.

The real-world execution of the following experiment is shown in Figure 7. The robot successfully tracked the gaze point until it became stationary, then gradually approached the target until reaching the pre-defined stopping volume. Figure 8a shows that the Tool Center Point (TCP), which represents the robot’s end-effector, successfully tracked the rectangular path defined by the gaze movement. However, the results also highlight the presence of noise in the gaze signal. A detailed examination of the x and y trajectory components in Figure 8b confirmed some noise, especially on the *y*-axis, along with an average delay of approximately 1 s.

**Figure 9:**
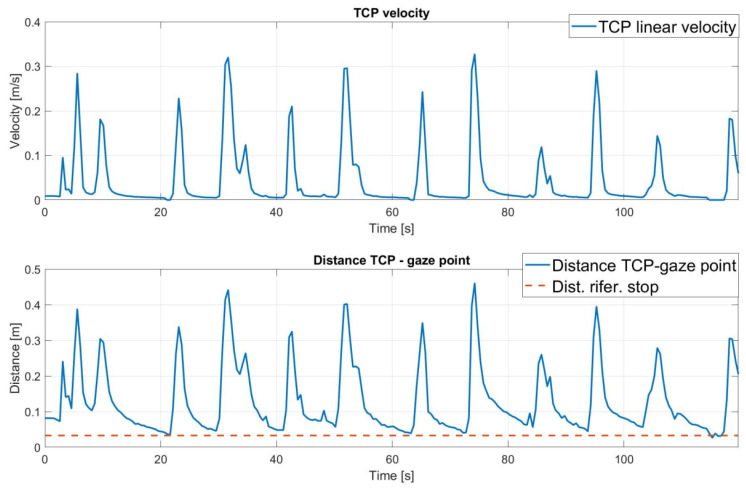
Norm of the end-effector linear velocity over time and the related distance between the gaze point and end-effector.

The analysis of the distance between the TCP and the target over the duration of the experiment in Figure 9 shows how the robot is able to approach the target. Observing the magnitude of the velocity, it can also be seen that the approach of the end-effector to the gaze point is gradual, until it stops. This is due to the choice of the bell function, which is particularly suitable for human–robot collaboration.

### 4.2. Filtered Gaze Signal with a Size of 5

**Figure 10:**
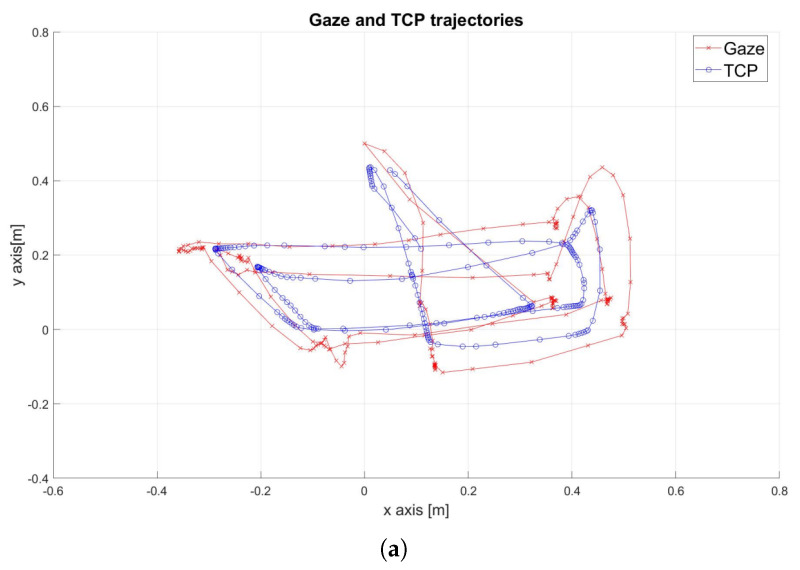

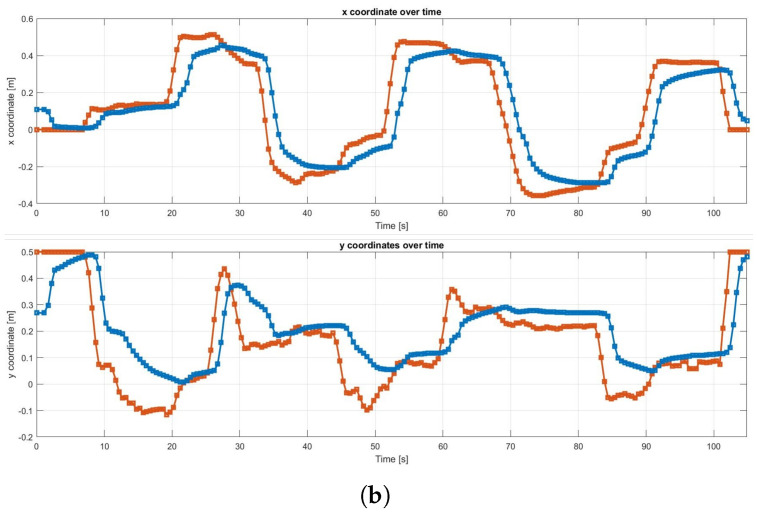
Filtered gaze signal with filter size 5, where gaze is represented in red and the end-effector is represented in blue. (**a**) Trajectories in the x–y plane. (**b**) Trajectories along the x and y axes over time.

In the second test, the gaze signal was filtered using a moving mean filter with a window size of 5. The robot effectively tracked the gaze point until it became stationary, and a reduction in signal noise was observed, particularly along the *x*-axis. However, a time delay of approximately 3 s was measured between the gaze input and the end-effector response. The results can be seen in Figure 10.

### 4.3. Filtered Gaze Signal with a Size of 10

**Figure 11:**
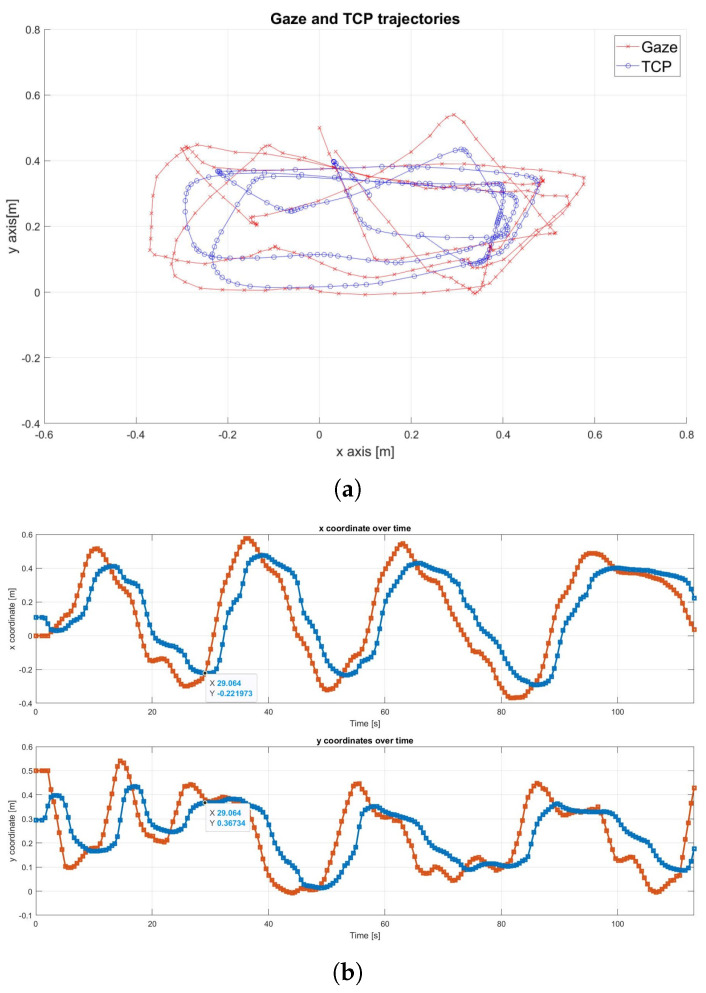
Filtered gaze signal with filter size 10, where gaze is represented in red and the end-effector is represented in blue. (**a**) Trajectories in the x–y plane. (**b**) Trajectories along the x and y axes over time.

The signal was further analyzed using a moving mean filter with an increased window size of 10, resulting in a delay of approximately 4 s, as shown in Figure 11. Additionally, the filtering occasionally caused the robot to miss specific points, particularly along the *y*-axis, leading to inconsistent tracking and premature transitions to subsequent targets. An example of this can be observed at 29 s.

### 4.4. Discussion

Among the three tests conducted, the filter with a window size of 10 proved to be the least favorable option; while it effectively eliminated noise, producing a smooth signal, it also introduced a significant delay, causing deviations in the robot’s gaze tracking. This effect is due to the imposed velocity profile, as shown in Figure 3. In the original signal, the sudden increase in distance between the end-effector and the target demands a higher velocity, in accordance with the predefined velocity curve. In contrast, the filtered signals, particularly the moving window filter with a size of 10, result in a smoother distance variation. As a consequence, the required velocity is lower in the initial phase, leading to a greater delay in reaching the reference position. Given the real-time application requirements, using the original gaze signal emerged as the optimal choice, outperforming the filter with a window size of 5. A detailed analysis of the graphs indicates that the end-effector tracked the gaze more accurately in this scenario. From a collaborative perspective, the original gaze signal was also preferred, due to its superior responsiveness to gaze movements.

The analyses presented focus on the robot’s ability to follow the input signal according to the provided reference. However, as reported in [23], OpenFace 2.0 exhibits a mean angular error of approximately 9 degrees in gaze direction estimation, which significantly affects the accuracy of the generated input and the identification of the correct gaze direction. According to the UR5 user manual [35], the robot’s repeatability is specified as ±0.1 mm.

## 5. Future Works

Future work will focus on several key aspects. Depending on the application, a crucial step in enabling autonomous robotic action is the integration of gripper control once the desired target position is reached. Preliminary tests have been conducted using a basic approach, where the gripper closes after a fixed delay following the end-effector’s movement. However, this strategy was excluded from the main discussion due to its oversimplification. Notably, gripper control should distinguish between closing and opening actions. In particular, opening the gripper typically assumes that an object has been grasped, and its release should be explicitly confirmed by the operator. Thus, the gripper should be activated through an auxiliary external signal.

Another issue is the management of unintended gaze shifts that can arise in situations where the user looks in a direction not meant to indicate intent. Future studies should investigate the extent to which such gaze errors are naturally filtered by the algorithm’s responsiveness, and whether reliable cues can be identified to distinguish them from intentional control signals.

Additionally, while, in this work, the target point moves on a plane, expanding it to support three-dimensional movement would significantly enhance its applicability in more complex manipulation tasks.

Finally, although the present work focuses on gaze-only control, future research could explore the integration of multiple input modalities. Gaze offers valuable anticipatory information, but its limitations as a standalone communication channel could be mitigated by combining it with other control mechanisms. Multimodal interaction could improve overall efficiency and bring human–robot collaboration closer to human–human coordination. A possible solution could involve the implementation of a depth camera, enabling the skeleton tracking of the operator and the integration of gesture recognition, as seen in [4].

## 6. Conclusions

This study examines the implementation of gaze tracking to enhance the efficiency of human–machine interaction, serving as a foundation for future developments aimed at integrating robot control through gaze tracking. The main gaze-tracking methodologies and their classifications were explored, along with a comparison of the technologies available on the market. The open-source software OpenFace 2.0 was selected for gaze-signal processing and integrated into a control framework based on MATLAB to manage the position of a manipulator in a two-dimensional space. The algorithm, with a low computational cost and latency, enables the real-time control of the cobot, and laboratory experiments to validate its applicability were performed.

Three scenarios were studied: the first with the raw gaze signal, the second with a moving average filter of size 5, and the third with the same filter of size 10. The experiments aimed to find a trade-off between latency and noise in the reference signal for the end-effector position. Although filtering made the signal smoother, the resulting latency was considered excessive, compromising the manipulator’s efficiency at following the reference. Despite the noise, the original signal was preferred for its higher responsiveness.

The gaze-processing system exhibits a mean angular error of approximately 9 degrees. Although, depending on the application, greater accuracy may be needed, the choice of OpenFace results from a deliberate trade-off, in which open-source software and non-wearable systems were preferred. Notably, improved performance could be achieved by replacing the gaze-acquisition module without requiring modifications to the main control architecture. The main limitations of the current system include the target gaze point being constrained to a plane, the lack of a dedicated gripper control mechanism, and the absence of a strategy to distinguish between gaze patterns that precede an action and accidental glances in non-operational directions. Moreover, since the main processing delay is caused by the MATLAB CSV reading function, implementing real-time data exchange through a different software solution could significantly improve time efficiency.

In conclusion, despite the simplicity of the control structure and its limitations, the results were assessed as satisfactory, suggesting that further refinement and adaptation to specific tasks could yield promising developments. Future research will focus on applying the gaze-tracking system presented to improve hand-over in collaborative robotics.

## Figures and Tables

**Table 1:** Comparison between several eye tracking systems, considering different features.

Gaze-Tracking System	Sampling Rate [Hz]	Accuracy [Degree]	Operating Distance [m]	Output	Programming Language	Open Source	Related Hardware
GazeTracking	Variable	Undisclosed	30–60	Gaze direction, pupil position	Python	Yes	Camera
OpenFace 2.0	Variable	9 (mean error)	30–100	Gaze direction, eye landmarks	MATLAB, C++, Python	Yes	Camera
GazeSense	10–90	1.5	30–80	Gaze direction, pupil origin	Python, C++	No	Camera
Pupil Core	30 (maximum)	0.6	Head-mounted	Gaze vector, pupil metrics	C, C++	No	Wearable device
GlassGaze	Variable	0.6	45–90	Gaze vector, pupil metric	Python, C++	Yes	Google Glass
Tobii Pro Glasses 3	50 or 100	0.6	Head-mounted	2D–3D gaze, gaze origin, gaze direction	Python, Javascript, HTML	Yes	Wearable glasses
Tobii Pro Spark	33 or 60	0.45	45–90	Gaze origin, gaze direction	Python, MATLAB, C	No	Dedicated camera
SMI Eye Tracking HMD	60	0.5–1	Head-mounted	Gaze vector, pupil metrics	Python, C++	No	Oculus Rift DK2
Smart Eye Pro	60–120	0.5–5	30–300	Eye position, eye gaze, eye metrics	Dedicated software	No	Dedicated camera

**Table 2:** Software and hardware set-ups used for the experiments.

Software Setup	
Operating System	Windows 10 Pro (22H2)
Gaze Tracking	OpenFace 2.2.0
Data Processing	MATLAB 2023a
UR5 Software	Polyscope 3.5
**Hardware Specifications**	
Processor	Intel Core i7-6700
RAM	32 GB
Graphics Card	NVIDIA GTX 970

## Data Availability

The data can be accessed upon request to the corresponding author.

## Supplementary Materials

The following supporting information can be downloaded at: https://www.mdpi.com/article/10.3390/s25103103/s1, Video S1: Real World Experiment.

## Abbreviations

The following abbreviations are used in this manuscript:

COBOT	Collaborative Robot
REGT	Remote Eye Gaze Tracker
D-H	Denavit-Hartenberg
TCP	Tool Center Point
